# Nigral Neurons Degenerating in Parkinson's Disease Express the Angiotensin Receptor Type 1 Gene

**DOI:** 10.1002/mds.29137

**Published:** 2022-06-29

**Authors:** Jose L. Labandeira‐Garcia, Juan A. Parga

**Affiliations:** ^1^ Laboratory of Cellular and Molecular Neurobiology of Parkinson's Disease, Research Center for Molecular Medicine and Chronic Diseases (CIMUS), IDIS University of Santiago de Compostela Santiago de Compostela Spain

## Abstract

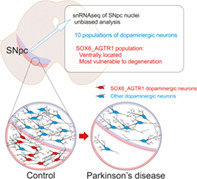

© 2022 The Authors. *Movement Disorders* published by Wiley Periodicals LLC on behalf of International Parkinson and Movement Disorder Society

Kamath T , Abdulraouf A , Burris SJ , et al. Single‐cell genomic profiling of human dopamine neurons identifies a population that selectively degenerates in Parkinson's disease. Nat Neurosci 2022;25(5):588–595.3551351510.1038/s41593-022-01061-1PMC9076534

The loss of dopaminergic neurons in the substantia nigra pars compacta (SNpc) is the neuropathological hallmark of Parkinson's disease (PD). However, neurons in the ventral tier of the SNpc are more vulnerable than those in the dorsal tier. The mechanism responsible for differences in vulnerability of dopaminergic neurons is a central and long‐standing question in the field of PD, because the answer may lead to major therapeutic advances. The heterogeneous composition of the SNpc neuron population has been further demonstrated with new single‐cell genomic methods.

Kamath et al^1^ used single‐nucleus RNA sequencing and unbiased clustering analysis of human SNpc dopaminergic neurons to define 10 transcriptionally distinct subpopulations, observing similar results in different mammalian species. One population, characterized by high levels of expression of both SOX6 and the angiotensin receptor type 1 (AGTR1) genes, was specifically located in the ventral tier of the SNpc of healthy control subjects, where neurodegeneration is more prominent in PD. In addition, the SOX6_AGTR1 subpopulation showed the largest loss of neurons when comparing patients with PD or Lewy body dementia with control subjects, and AGTR1 expression correlated with susceptibility to neurodegeneration. SNpc dopaminergic neurons, but not other cell populations, showed an enrichment of genes associated with increased PD risk, suggesting a cell‐intrinsic mechanism. This enrichment appears related to the SOX6_AGTR1 neuron subtype, which showed the largest increase. Moreover, the SOX6_AGTR1 subpopulation expressed lower levels of genes regulated by the dopaminergic‐differentiation transcription factor LMX1A, but overexpressed TP53 and NR2F2, which are involved in dopaminergic neurodegeneration. The causal relationship between gene expression and dopaminergic vulnerability has not been clarified.

However, the findings are consistent with those of previous studies in PD models suggesting a major role of the brain renin‐angiotensin system (RAS) in promoting dopaminergic neurodegeneration by activation of the pro‐oxidative and proinflammatory AGTR1.^2^, ^3^ Furthermore, an intracellular RAS was identified in mitochondria and nuclei of dopaminergic neurons, including other RAS components such as AGTR2 and MAS receptors that may counteract the deleterious effects of AGTR1 activation.^4^ Recent clinical studies also support a neuroprotective effect of AGTR1 inhibitors.^3^, ^5^ The findings of Kamath et al^1^ further encourage the development of prodromal clinical trials for angiotensin receptor blockers (ARBS) that can cross the blood‐brain barrier or for molecules inhibiting AGTR1 effects, including those acting on RAS regulatory components (AGTR2, MAS) that counteract AGTR1 activation. Furthermore, their results may also improve PD cell therapies by neuroprotecting or selectively replacing the most vulnerable cells.

## Data Availability

Not applicable (hot topic)
